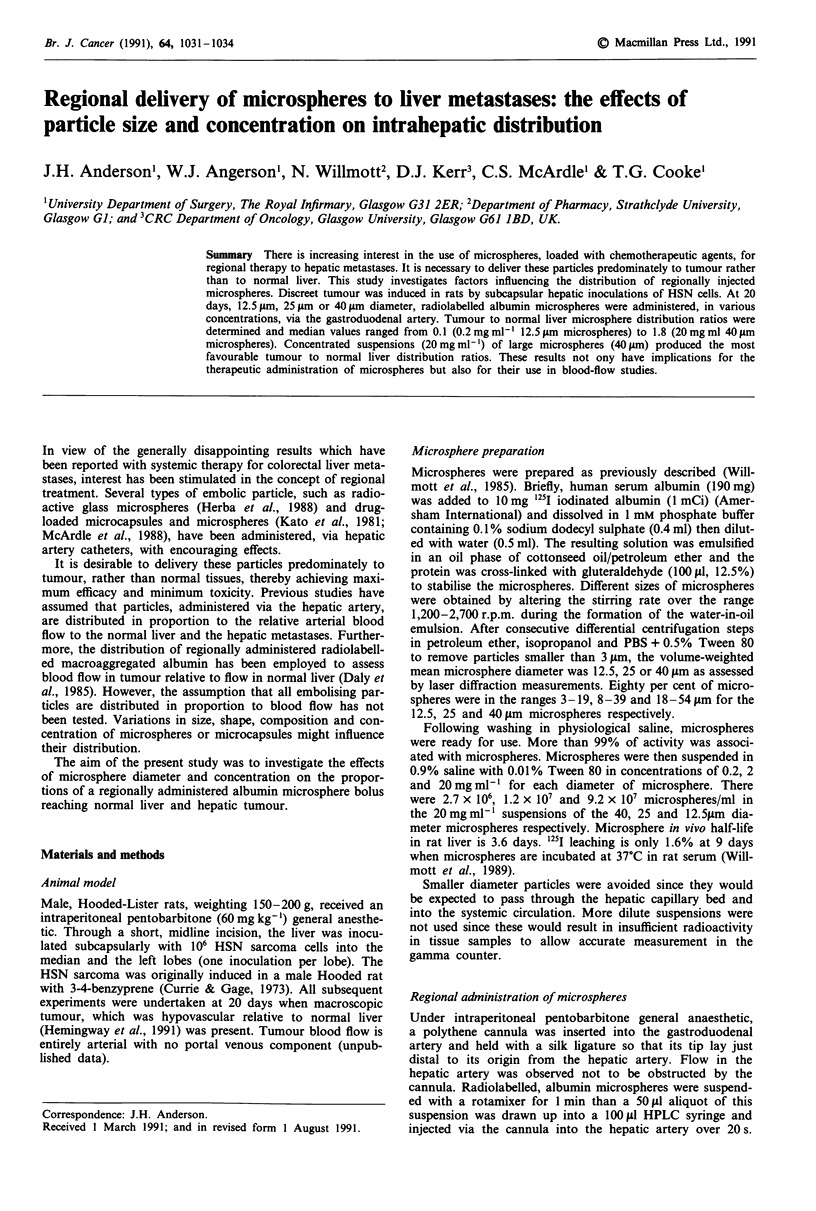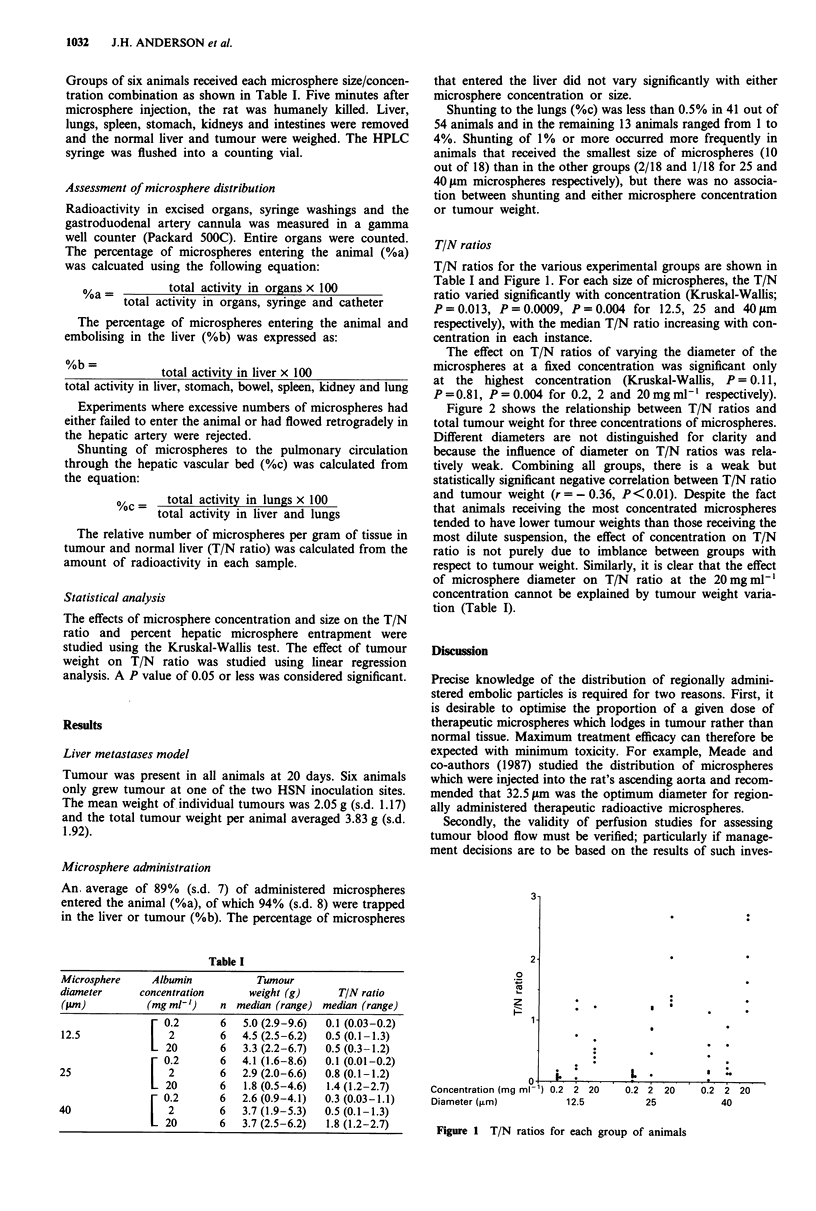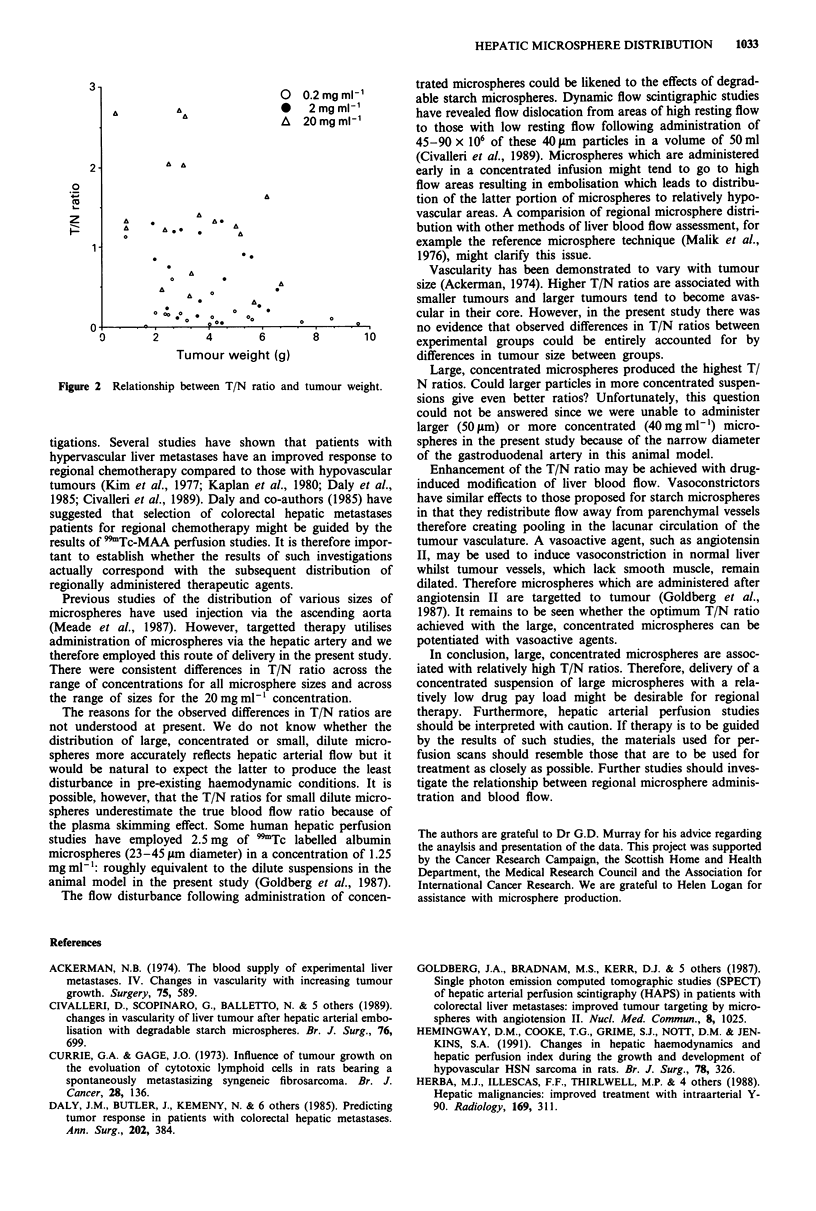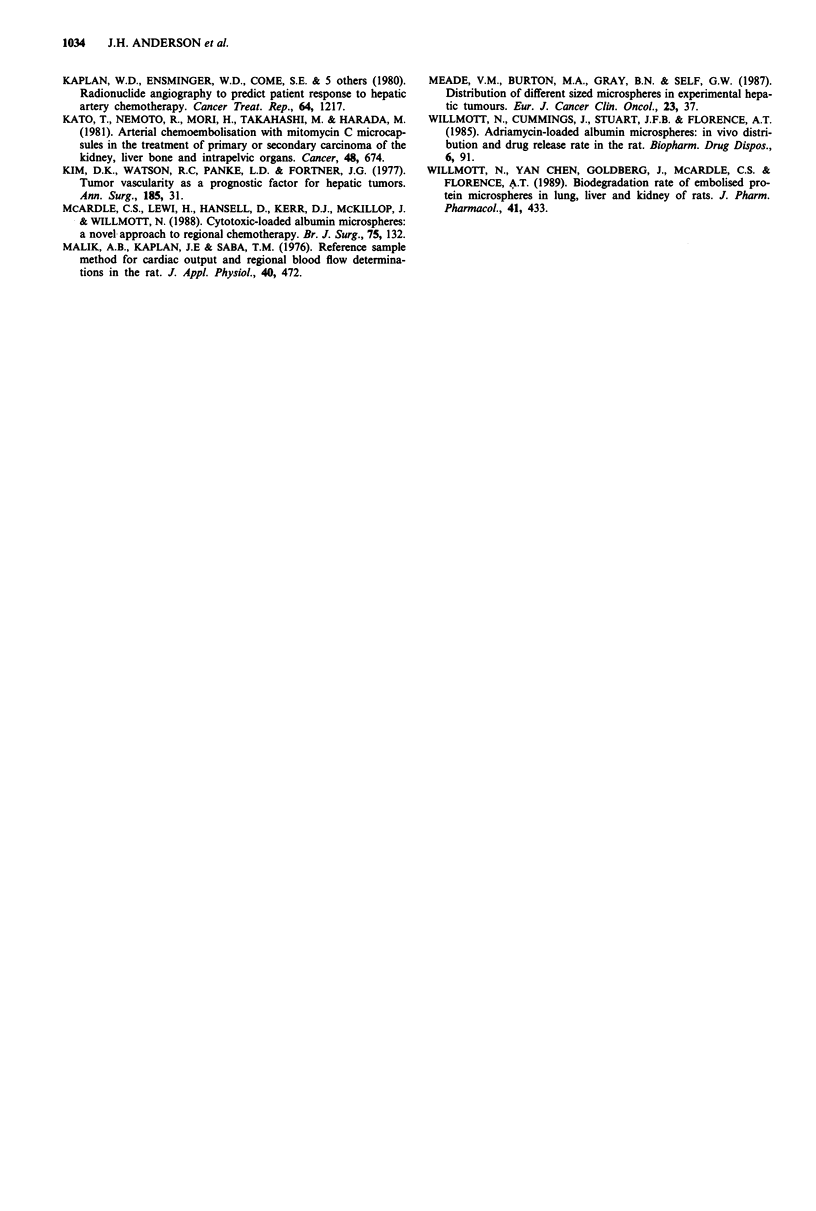# Regional delivery of microspheres to liver metastases: the effects of particle size and concentration on intrahepatic distribution.

**DOI:** 10.1038/bjc.1991.459

**Published:** 1991-12

**Authors:** J. H. Anderson, W. J. Angerson, N. Willmott, D. J. Kerr, C. S. McArdle, T. G. Cooke

**Affiliations:** University Department of Surgery, Royal Infirmary, Glasgow, UK.

## Abstract

There is increasing interest in the use of microspheres, loaded with chemotherapeutic agents, for regional therapy to hepatic metastases. It is necessary to deliver these particles predominately to tumour rather than to normal liver. This study investigates factors influencing the distribution of regionally injected microspheres. Discreet tumour was induced in rats by subcapsular hepatic inoculations of HSN cells. At 20 days, 12.5 microns, 25 microns or 40 microns diameter, radiolabelled albumin microspheres were administered, in various concentrations, via the gastroduodenal artery. Tumour to normal liver microsphere distribution ratios were determined and median values ranged from 0.1 (0.2 mg ml-1 12.5 microns microspheres) to 1.8 (20 mg ml 40 microns microspheres). Concentrated suspensions (20 mg ml-1) of large microspheres (40 microns) produced the most favourable tumour to normal liver distribution ratios. These results not only have implications for the therapeutic administration of microspheres but also for their use in blood-flow studies.


© Macmillan Press Ltd., 1991

### Regional delivery of microspheres to liver metastases: the effects of particle size and concentration on intrahepatic distribution

J.H. Anderson[1], W.J. Angerson[1], N. Willmott[2], D.J. Kerr[3], C.S. McArdle[1] & T.G. Cooke[1]

[1]*University Department of Surgery, The Royal Infirmary, Glasgow G31 2ER;* [2]*Department of Pharmacy, Strathclyde University, Glasgow G1; and* [3]*CRC Department of Oncology, Glasgow University, Glasgow G61 1BD, UK.*

**Summary** There is increasing interest in the use of microspheres, loaded with chemotherapeutic agents, for regional therapy to hepatic metastases. It is necessary to deliver these particles predominately to tumour rather than to normal liver. This study investigates factors influencing the distribution of regionally injected microspheres. Discreet tumour was induced in rats by subcapsular hepatic inoculations of HSN cells. At 20 days, 12.5 μm, 25 μm or 40 μm diameter, radiolabelled albumin microspheres were administered, in various concentrations, via the gastroduodenal artery. Tumour to normal liver microsphere distribution ratios were determined and median values ranged from 0.1 (0.2 mg ml$^{-1}$ 12.5 μm microspheres) to 1.8 (20 mg ml 40 μm microspheres). Concentrated suspensions (20 mg ml$^{-1}$) of large microspheres (40 μm) produced the most favourable tumour to normal liver distribution ratios. These results not ony have implications for the therapeutic administration of microspheres but also for their use in blood-flow studies.

In view of the generally disappointing results which have been reported with systemic therapy for colorectal liver metastases, interest has been stimulated in the concept of regional treatment. Several types of embolic particle, such as radioactive glass microspheres (Herba *et al.*, 1988) and drug-loaded microcapsules and microspheres (Kato *et al.*, 1981; McArdle *et al.*, 1988), have been administered, via hepatic artery catheters, with encouraging effects.

It is desirable to delivery these particles predominately to tumour, rather than normal tissues, thereby achieving maximum efficacy and minimum toxicity. Previous studies have assumed that particles, administered via the hepatic artery, are distributed in proportion to the relative arterial blood flow to the normal liver and the hepatic metastases. Furthermore, the distribution of regionally administered radiolabelled macroaggregated albumin has been employed to assess blood flow in tumour relative to flow in normal liver (Daly *et al.*, 1985). However, the assumption that all embolising particles are distributed in proportion to blood flow has not been tested. Variations in size, shape, composition and concentration of microspheres or microcapsules might influence their distribution.

The aim of the present study was to investigate the effects of microsphere diameter and concentration on the proportions of a regionally administered albumin microsphere bolus reaching normal liver and hepatic tumour.

### Materials and methods

### *Animal model*

Male, Hooded-Lister rats, weighting 150-200 g, received an intraperitoneal pentobarbitone (60 mg kg$^{-1}$) general anesthetic. Through a short, midline incision, the liver was inoculated subcapsularly with $10^6$ HSN sarcoma cells into the median and the left lobes (one inoculation per lobe). The HSN sarcoma was originally induced in a male Hooded rat with 3-4-benzyprene (Currie & Gage, 1973). All subsequent experiments were undertaken at 20 days when macroscopic tumour, which was hypovascular relative to normal liver (Hemingway *et al.*, 1991) was present. Tumour blood flow is entirely arterial with no portal venous component (unpublished data).

### *Microsphere preparation*

Microspheres were prepared as previously described (Willmott *et al.*, 1985). Briefly, human serum albumin (190 mg) was added to 10 mg $^{125}$I iodinated albumin (1 mCi) (Amersham International) and dissolved in 1 mM phosphate buffer containing 0.1% sodium dodecyl sulphate (0.4 ml) then diluted with water (0.5 ml). The resulting solution was emulsified in an oil phase of cottonseed oil/petroleum ether and the protein was cross-linked with gluteraldehyde (100 μl, 12.5%) to stabilise the microspheres. Different sizes of microspheres were obtained by altering the stirring rate over the range 1,200-2,700 r.p.m. during the formation of the water-in-oil emulsion. After consecutive differential centrifugation steps in petroleum ether, isopropanol and PBS + 0.5% Tween 80 to remove particles smaller than 3 μm, the volume-weighted mean microsphere diameter was 12.5, 25 or 40 μm as assessed by laser diffraction measurements. Eighty per cent of microspheres were in the ranges 3-19, 8-39 and 18-54 μm for the 12.5, 25 and 40 μm microspheres respectively.

Following washing in physiological saline, microspheres were ready for use. More than 99% of activity was associated with microspheres. Microspheres were then suspended in 0.9% saline with 0.01% Tween 80 in concentrations of 0.2, 2 and 20 mg ml$^{-1}$ for each diameter of microsphere. There were $2.7 \times 10^6$, $1.2 \times 10^7$ and $9.2 \times 10^7$ microspheres/ml in the 20 mg ml$^{-1}$ suspensions of the 40, 25 and 12.5μm diameter microspheres respectively. Microsphere *in vivo* half-life in rat liver is 3.6 days. $^{125}$I leaching is only 1.6% at 9 days when microspheres are incubated at 37°C in rat serum (Willmott *et al.*, 1989).

Smaller diameter particles were avoided since they would be expected to pass through the hepatic capillary bed and into the systemic circulation. More dilute suspensions were not used since these would result in insufficient radioactivity in tissue samples to allow accurate measurement in the gamma counter.

### *Regional administration of microspheres*

Under intraperitoneal pentobarbitone general anaesthetic, a polythene cannula was inserted into the gastroduodenal artery and held with a silk ligature so that its tip lay just distal to its origin from the hepatic artery. Flow in the hepatic artery was observed not to be obstructed by the cannula. Radiolabelled, albumin microspheres were suspended with a rotamixer for 1 min than a 50 μl aliquot of this suspension was drawn up into a 100 μl HPLC syringe and injected via the cannula into the hepatic artery over 20 s.

Correspondence: J.H. Anderson.
Received 1 March 1991; and in revised form 1 August 1991.

Groups of six animals received each microsphere size/concentration combination as shown in Table I. Five minutes after microsphere injection, the rat was humanely killed. Liver, lungs, spleen, stomach, kidneys and intestines were removed and the normal liver and tumour were weighed. The HPLC syringe was flushed into a counting vial.

*Assessment of microsphere distribution*

Radioactivity in excised organs, syringe washings and the gastroduodenal artery cannula was measured in a gamma well counter (Packard 500C). Entire organs were counted. The percentage of microspheres entering the animal (%a) was calcuated using the following equation:

$$\%a = \frac{\text{total activity in organs} \times 100}{\text{total activity in organs, syringe and catheter}}$$

The percentage of microspheres entering the animal and embolising in the liver (%b) was expressed as:

$$\%b = \frac{\text{total activity in liver} \times 100}{\text{total activity in liver, stomach, bowel, spleen, kidney and lung}}$$

Experiments where excessive numbers of microspheres had either failed to enter the animal or had flowed retrogradely in the hepatic artery were rejected.

Shunting of microspheres to the pulmonary circulation through the hepatic vascular bed (%c) was calculated from the equation:

$$\%c = \frac{\text{total activity in lungs} \times 100}{\text{total activity in liver and lungs}}$$

The relative number of microspheres per gram of tissue in tumour and normal liver (T/N ratio) was calculated from the amount of radioactivity in each sample.

*Statistical analysis*

The effects of microsphere concentration and size on the T/N ratio and percent hepatic microsphere entrapment were studied using the Kruskal-Wallis test. The effect of tumour weight on T/N ratio was studied using linear regression analysis. A $P$ value of 0.05 or less was considered significant.

### Results

*Liver metastases model*

Tumour was present in all animals at 20 days. Six animals only grew tumour at one of the two HSN inoculation sites. The mean weight of individual tumours was 2.05 g (s.d. 1.17) and the total tumour weight per animal averaged 3.83 g (s.d. 1.92).

*Microsphere administration*

An average of 89% (s.d. 7) of administered microspheres entered the animal (%a), of which 94% (s.d. 8) were trapped in the liver or tumour (%b). The percentage of microspheres that entered the liver did not vary significantly with either microsphere concentration or size.

Shunting to the lungs (%c) was less than 0.5% in 41 out of 54 animals and in the remaining 13 animals ranged from 1 to 4%. Shunting of 1% or more occurred more frequently in animals that received the smallest size of microspheres (10 out of 18) than in the other groups (2/18 and 1/18 for 25 and 40 µm microspheres respectively), but there was no association between shunting and either microsphere concentration or tumour weight.

*T/N ratios*

T/N ratios for the various experimental groups are shown in Table I and Figure 1. For each size of microspheres, the T/N ratio varied significantly with concentration (Kruskal-Wallis; $P = 0.013$, $P = 0.0009$, $P = 0.004$ for 12.5, 25 and 40 µm respectively), with the median T/N ratio increasing with concentration in each instance.

The effect on T/N ratios of varying the diameter of the microspheres at a fixed concentration was significant only at the highest concentration (Kruskal-Wallis, $P = 0.11$, $P = 0.81$, $P = 0.004$ for 0.2, 2 and 20 mg ml$^{-1}$ respectively).

Figure 2 shows the relationship between T/N ratios and total tumour weight for three concentrations of microspheres. Different diameters are not distinguished for clarity and because the influence of diameter on T/N ratios was relatively weak. Combining all groups, there is a weak but statistically significant negative correlation between T/N ratio and tumour weight ($r = -0.36$, $P < 0.01$). Despite the fact that animals receiving the most concentrated microspheres tended to have lower tumour weights than those receiving the most dilute suspension, the effect of concentration on T/N ratio is not purely due to imblance between groups with respect to tumour weight. Similarly, it is clear that the effect of microsphere diameter on T/N ratio at the 20 mg ml$^{-1}$ concentration cannot be explained by tumour weight variation (Table I).

### Discussion

Precise knowledge of the distribution of regionally administered embolic particles is required for two reasons. First, it is desirable to optimise the proportion of a given dose of therapeutic microspheres which lodges in tumour rather than normal tissue. Maximum treatment efficacy can therefore be expected with minimum toxicity. For example, Meade and co-authors (1987) studied the distribution of microspheres which were injected into the rat's ascending aorta and recommended that 32.5 µm was the optimum diameter for regionally administered therapeutic radioactive microspheres.

Secondly, the validity of perfusion studies for assessing tumour blood flow must be verified; particularly if management decisions are to be based on the results of such inves-

**Table I**

| Microsphere diameter (µm) | Albumin concentration (mg ml$^{-1}$) | n | Tumour weight (g) median (range) | T/N ratio median (range) |
|---|---|---|---|---|
| 12.5 | 0.2 | 6 | 5.0 (2.9–9.6) | 0.1 (0.03–0.2) |
| | 2 | 6 | 4.5 (2.5–6.2) | 0.5 (0.1–1.3) |
| | 20 | 6 | 3.3 (2.2–6.7) | 0.5 (0.3–1.2) |
| 25 | 0.2 | 6 | 4.1 (1.6–8.6) | 0.1 (0.01–0.2) |
| | 2 | 6 | 2.9 (2.0–6.6) | 0.8 (0.1–1.2) |
| | 20 | 6 | 1.8 (0.5–4.6) | 1.4 (1.2–2.7) |
| 40 | 0.2 | 6 | 2.6 (0.9–4.1) | 0.3 (0.03–1.1) |
| | 2 | 6 | 3.7 (1.9–5.3) | 0.5 (0.1–1.3) |
| | 20 | 6 | 3.7 (2.5–6.2) | 1.8 (1.2–2.7) |

**Figure 1** T/N ratios for each group of animals

**Figure 2** Relationship between T/N ratio and tumour weight.

tigations. Several studies have shown that patients with hypervascular liver metastases have an improved response to regional chemotherapy compared to those with hypovascular tumours (Kim *et al.*, 1977; Kaplan *et al.*, 1980; Daly *et al.*, 1985; Civalleri *et al.*, 1989). Daly and co-authors (1985) have suggested that selection of colorectal hepatic metastases patients for regional chemotherapy might be guided by the results of $^{99m}$Tc-MAA perfusion studies. It is therefore important to establish whether the results of such investigations actually correspond with the subsequent distribution of regionally administered therapeutic agents.

Previous studies of the distribution of various sizes of microspheres have used injection via the ascending aorta (Meade *et al.*, 1987). However, targetted therapy utilises administration of microspheres via the hepatic artery and we therefore employed this route of delivery in the present study. There were consistent differences in T/N ratio across the range of concentrations for all microsphere sizes and across the range of sizes for the 20 mg ml$^{-1}$ concentration.

The reasons for the observed differences in T/N ratios are not understood at present. We do not know whether the distribution of large, concentrated or small, dilute microspheres more accurately reflects hepatic arterial flow but it would be natural to expect the latter to produce the least disturbance in pre-existing haemodynamic conditions. It is possible, however, that the T/N ratios for small dilute microspheres underestimate the true blood flow ratio because of the plasma skimming effect. Some human hepatic perfusion studies have employed 2.5 mg of $^{99m}$Tc labelled albumin microspheres (23–45 μm diameter) in a concentration of 1.25 mg ml$^{-1}$: roughly equivalent to the dilute suspensions in the animal model in the present study (Goldberg *et al.*, 1987).

The flow disturbance following administration of concentrated microspheres could be likened to the effects of degradable starch microspheres. Dynamic flow scintigraphic studies have revealed flow dislocation from areas of high resting flow to those with low resting flow following administration of 45–90 × 10$^{6}$ of these 40 μm particles in a volume of 50 ml (Civalleri *et al.*, 1989). Microspheres which are administered early in a concentrated infusion might tend to go to high flow areas resulting in embolisation which leads to distribution of the latter portion of microspheres to relatively hypovascular areas. A comparision of regional microsphere distribution with other methods of liver blood flow assessment, for example the reference microsphere technique (Malik *et al.*, 1976), might clarify this issue.

Vascularity has been demonstrated to vary with tumour size (Ackerman, 1974). Higher T/N ratios are associated with smaller tumours and larger tumours tend to become avascular in their core. However, in the present study there was no evidence that observed differences in T/N ratios between experimental groups could be entirely accounted for by differences in tumour size between groups.

Large, concentrated microspheres produced the highest T/N ratios. Could larger particles in more concentrated suspensions give even better ratios? Unfortunately, this question could not be answered since we were unable to administer larger (50 μm) or more concentrated (40 mg ml$^{-1}$) microspheres in the present study because of the narrow diameter of the gastroduodenal artery in this animal model.

Enhancement of the T/N ratio may be achieved with drug-induced modification of liver blood flow. Vasoconstrictors have similar effects to those proposed for starch microspheres in that they redistribute flow away from parenchymal vessels therefore creating pooling in the lacunar circulation of the tumour vasculature. A vasoactive agent, such as angiotensin II, may be used to induce vasoconstriction in normal liver whilst tumour vessels, which lack smooth muscle, remain dilated. Therefore microspheres which are administered after angiotensin II are targetted to tumour (Goldberg *et al.*, 1987). It remains to be seen whether the optimum T/N ratio achieved with the large, concentrated microspheres can be potentiated with vasoactive agents.

In conclusion, large, concentrated microspheres are associated with relatively high T/N ratios. Therefore, delivery of a concentrated suspension of large microspheres with a relatively low drug pay load might be desirable for regional therapy. Furthermore, hepatic arterial perfusion studies should be interpreted with caution. If therapy is to be guided by the results of such studies, the materials used for perfusion scans should resemble those that are to be used for treatment as closely as possible. Further studies should investigate the relationship between regional microsphere administration and blood flow.

The authors are grateful to Dr G.D. Murray for his advice regarding the anaylsis and presentation of the data. This project was supported by the Cancer Research Campaign, the Scottish Home and Health Department, the Medical Research Council and the Association for International Cancer Research. We are grateful to Helen Logan for assistance with microsphere production.

### References

ACKERMAN, N.B. (1974). The blood supply of experimental liver metastases. IV. Changes in vascularity with increasing tumour growth. *Surgery*, **75**, 589.

CIVALLERI, D., SCOPINARO, G., BALLETTO, N. & 5 others (1989). changes in vascularity of liver tumour after hepatic arterial embolisation with degradable starch microspheres. *Br. J. Surg.*, **76**, 699.

CURRIE, G.A. & GAGE, J.O. (1973). Influence of tumour growth on the evoluation of cytotoxic lymphoid cells in rats bearing a spontaneously metastasizing syngeneic fibrosarcoma. *Br. J. Cancer*, **28**, 136.

DALY, J.M., BUTLER, J., KEMENY, N. & 6 others (1985). Predicting tumor response in patients with colorectal hepatic metastases. *Ann. Surg.*, **202**, 384.

GOLDBERG, J.A., BRADNAM, M.S., KERR, D.J. & 5 others (1987). Single photon emission computed tomographic studies (SPECT) of hepatic arterial perfusion scintigraphy (HAPS) in patients with colorectal liver metastases: improved tumour targeting by microspheres with angiotension II. *Nucl. Med. Commun.*, **8**, 1025.

HEMINGWAY, D.M., COOKE, T.G., GRIME, S.J., NOTT, D.M. & JENKINS, S.A. (1991). Changes in hepatic haemodynamics and hepatic perfusion index during the growth and development of hypovascular HSN sarcoma in rats. *Br. J. Surg.*, **78**, 326.

HERBA, M.J., ILLESCAS, F.F., THIRLWELL, M.P. & 4 others (1988). Hepatic malignancies: improved treatment with intraarterial Y-90. *Radiology*, **169**, 311.

KAPLAN, W.D., ENSMINGER, W.D., COME, S.E. & 5 others (1980). Radionuclide angiography to predict patient response to hepatic artery chemotherapy. *Cancer Treat. Rep.*, **64**, 1217.

KATO, T., NEMOTO, R., MORI, H., TAKAHASHI, M. & HARADA, M. (1981). Arterial chemoembolisation with mitomycin C microcapsules in the treatment of primary or secondary carcinoma of the kidney, liver bone and intrapelvic organs. *Cancer*, **48**, 674.

KIM, D.K., WATSON, R.C, PANKE, L.D. & FORTNER, J.G. (1977). Tumor vascularity as a prognostic factor for hepatic tumors. *Ann. Surg.*, **185**, 31.

MCARDLE, C.S., LEWI, H., HANSELL, D., KERR, D.J., MCKILLOP, J. & WILLMOTT, N. (1988). Cytotoxic-loaded albumin microspheres: a novel approach to regional chemotherapy. *Br. J. Surg.*, **75**, 132.

MALIK, A.B., KAPLAN, J.E & SABA, T.M. (1976). Reference sample method for cardiac output and regional blood flow determinations in the rat. *J. Appl. Physiol.*, **40**, 472.

MEADE, V.M., BURTON, M.A., GRAY, B.N. & SELF, G.W. (1987). Distribution of different sized microspheres in experimental hepatic tumours. *Eur. J. Cancer Clin. Oncol.*, **23**, 37.

WILLMOTT, N., CUMMINGS, J., STUART, J.F.B. & FLORENCE, A.T. (1985). Adriamycin-loaded albumin microspheres: in vivo distribution and drug release rate in the rat. *Biopharm. Drug Dispos.*, **6**, 91.

WILLMOTT, N., YAN CHEN, GOLDBERG, J., MCARDLE, C.S. & FLORENCE, A.T. (1989). Biodegradation rate of embolised protein microspheres in lung, liver and kidney of rats. *J. Pharm. Pharmacol.*, **41**, 433.